# A new approach to enhance the performance of decision tree for classifying gene expression data

**DOI:** 10.1186/1753-6561-7-S7-S3

**Published:** 2013-12-20

**Authors:** Md Rafiul Hassan, Ramamohanarao Kotagiri

**Affiliations:** 1 Department of Information and Computer Science, King Fahd University of Petroleum and Minerals, Dhahran, Kingdom of Saudi Arabia; 2 Department of Computer Science and Software Engineering, The University of Melbourne, VIC 3110, Australia

## Abstract

**Background:**

Gene expression data classification is a challenging task due to the large dimensionality and very small number of samples. Decision tree is one of the popular machine learning approaches to address such classification problems. However, the existing decision tree algorithms use a single gene feature at each node to split the data into its child nodes and hence might suffer from poor performance specially when classifying gene expression dataset.

**Results:**

By using a new decision tree algorithm where, each node of the tree consists of more than one gene, we enhance the classification performance of traditional decision tree classifiers. Our method selects suitable genes that are combined using a linear function to form a derived composite feature. To determine the structure of the tree we use the area under the Receiver Operating Characteristics curve (AUC). Experimental analysis demonstrates higher classification accuracy using the new decision tree compared to the other existing decision trees in literature.

**Conclusion:**

We experimentally compare the effect of our scheme against other well known decision tree techniques. Experiments show that our algorithm can substantially boost the classification performance of the decision tree.

## Introduction

There are a lot of diseases available which needs to investigate more to understand them better. Due to lack of understanding of diseases e.g. breast cancer, often different outcome is shown for the same treatment applied to patients with similar clinical symptoms. Patient specific treatment could be one of the solutions to overcome this, however the varying outcome might be due to the limited knowledge about the relationship between treatment, disease development and clinical symptoms. The advent of gene expression data has opened up an opportunity to better understand diseases. However, to analyze the sheer amount of gene expression data is somehow complex and challenging due to the large dimension and small number of samples. The ultimate aim of analysis the data is to better diagnose and prognosticate diseases which in turn would provide an insight understanding the clinically relevant disease categories. Hence, an automated but simple computational technique is required to develop to classify diseases accurately using such high dimensional gene expression data. Among the existing machine learning techniques, decision tree is a well known and easy to understand classification technique [1,2]. Its construction cost scales well for many features and instances and it is easily interpretable and require few parameter settings. Unfortunately, not many studies are available that used decision tree to classify gene expression data. This may be attributed due to the poor performance because of the limitation of dealing with high dimension but small number of instances. In bioinformatics, one of the general goals is to apply computers for analysis of gene expression data and classify them to appropriate diseases/disease status accurately. In this paper we describe our own modest efforts towards this goal through developing a new decision tree.

Decision tree is typically induced by selecting the gene feature for a node that has the least impurity when compared to the other gene features in the dataset. The gene expression dataset at the node is split into its child nodes using the selected gene feature such that the impurity is reduced as far as possible. Thus there are two phases and two issues that need to be considered while inducing a decision tree given the gene expression dataset with class levels. The phases are described in brief here:

- **Node selection:** A decision tree is induced first by choosing a node as a root of the tree. For example, to classify the two types of lesion benign and malignant, we identified that the gene TFF3 can discriminate the patients with the least impurity. Hence, TFF3 gene is selected as root of the tree that divides the complete dataset into two or more subgroups with an aim to classify the dataset. The classification performance varies with varying choice of impurity measurement that would guide to induce the decision tree.

- **Splitting threshold:** Once the node is selected, the dataset needs to be partitioned by choosing an optimal threshold value of the selected node. For example, we divide the complete dataset into two groups where in one group TFF3 *>*0.54 (i.e., the splitting threshold = 0.54) and in another group TFF3 ≤ 0.54. And we achieve the best classification performance for the above mentioned threshold among the performances by choosing other threshold values. Thus, the optimal splitting threshold for the node TFF3 will be 0.54.

The issues in inducing decision tree are described in brief here:

- **Stopping criteria** In the process of inducing a decision tree the stopping criteria must be chosen to stop growing the tree at a suitable level, such that a better classification performance is achieved.

- **Labeling terminal nodes** Terminal/leaf nodes are those nodes at which point growing of the tree is stopped, i.e., terminal nodes do not have any children. Usually class label (e.g., benign or malignant) is decided based on the labeling of the terminal nodes.

Several techniques have been applied over time to measure the impurity and the most popular ones are the entropy-like uncertainty measures (i.e., gain ratio, information gain and gini index). Recently, Hossain *et al. *[3,4] developed a decision tree called *ROC-tree*, where Area Under Curve (AUC) is applied as an alternative to the entropy-like uncertainty to attain an accurate classification of gene expression data. However, like other existing decision trees in *ROC-tree*, each node is formed using a single gene feature. The feature that has the maximum AUC value with respect to the associated data is selected for a node. However, when more than one gene are combined using a linear function at each node of the tree, it can provide potentially even higher AUC value at each node compared to the single gene. Hence using multiple gene features for decision making at each node can improve performance substantially. In this paper we combined two genes together at each of the node of a decision tree to classify gene expression data. We call such trees as bi-variate decision trees. We further motivate with the following example:

**Example 1 ***Let us consider a gene expression dataset consisting of a large number of gene features A*_1_, *A*_2_, *A*_3_, ......, *A_m _(m is some large number). Assume that the highest AUC is achieved for the feature A*_200 _*which is 0.6. This feature having the highest AUC among the all gene features, is selected as the node of the ROC-tree. However, a linear combination of A*_450 _*and A*_29 _*(here, we consider a function to map the multiple gene features to a derived feature) provides an AUC of *0.75 *that is higher than the maximum AUC of any single gene feature. Instead of building a tree using A*_200 _*as a node decision variable we propose to use the linear combination of A*_450 _*and A*_29 _*as the node of the tree*.

As shown above, the limitation of *ROC-tree *is, it only uses one gene at one node. In this paper, to alleviate this problem, we consider more than one gene expression at each node. In order to use multiple gene features at each node, we use least square estimation (LSE) to map the multiple features to one derived feature. Which is used to estimate the AUC value as in *ROC-tree*. To split the dataset at the node we consider using a loss function known as Hinge-Rank-Loss [5]. Since, in this paper we restrict to use two gene features as a node to induce the decision tree, we call the new decision tree as bi-variate *ROC-tree *(*BVROC-tree*).

## Preliminaries

### The receiver operating characteristic Curve

A receiver operating characteristics (ROC) curve, first used in signal detection theory, is used to evaluate the discriminative performance of a binary classifier. This is achieved by plotting the curve of the *sensitivity *vs. (1 *− specificity*) for the binary classifier system by varying the discrimination threshold. The area under the ROC curve (AUC) can be computed using the trapezoidal integration. The maximum value of AUC can be 1 which indicates perfect classification whereas a value close to 0 indicates poor performance.

### ROC-tree

Previous work known as *ROC-tree *[3,4] has established the use of an ROC curve for node selection, to identify the discriminative features in the dataset and to induce a decision tree. First, the ROC curve is plotted for each of the pairs formed by each of the features and the class label. This means treating a single feature as a classifier and calculating the classification in terms of the *sensitivity *and *specificity *by varying the operating point. For each feature, the AUC is calculated and the feature with the highest AUC is selected for the node of the tree. The splitting threshold is chosen by taking each value of the selected feature from the dataset, and then attempting to classify based on a chosen value and calculating the misclassification rate for that value. The value with the minimum misclassification rate is finally chosen as the splitting threshold.

## Bi-variate ROC-tree: BVROC-tree

We now describe in more detail, the steps in our algorithm for building the *BVROC-tree*.

### Selection of more than one feature as node of the tree

In *ROC-tree *a single feature is selected for a node that provides the maximum AUC among the all other features. According to the following theorem, if we map any single feature to a derived feature using any monotonic function *f*(.) the area under curve is not affected.

**Theorem 1 ***The AUC*(*A*) = *AUC*(*f*(*A*)), *where, AUC(A) is area under curve using a single feature A and f*(.) *is any monotonic function*.

The implication of the theorem 1 is in building a bi-variate decision tree we do not need to consider mapping of any single feature. However, to use more than one feature at each node the set of features is mapped to a single feature using a linear function *f*(.) as in Eq. 1.

(1) $$Y^{\prime }={\vec{b}}^{T}*D_{s}$$

here, *Ds *= the dataset with several features selected from the training dataset *D*. $\vec{b}$ represents the co-efficient and $Y^{\prime }$ is the set of derived single feature values. The values of the co-efficient $\vec{b}$ are obtained by applying the least square estimation (LSE) formula.

Let us consider, *D_s _*= the dataset that contains all the values of the multiple selected features {*A_α_*, *A_β_*}. Thus, the values of $\vec{b}$ is obtained using Eq. 2.

(2) $$\vec{b}={CD}_{\text{s}}^{T}Y$$

where, $C={\left(D_{\text{S}}^{T}D_{\text{s}}\right)}^{-1}$ and $Y=\left(y_{1}\;y_{2}\;\ldots y_{m}\right)$, *m *= total number of data instances.

Initially, the ROC curve is plotted for each of the pairs formed by each of the features along with the class label and the corresponding AUC is computed. The feature that has the highest AUC is identified. Let us assume this feature is **A_s_**. This selected feature is then paired with each feature in the remaining feature set and the corresponding linear co-efficient is computed using LSE formula. For one such pair {**A_s_**, **A_p_**}, the co-efficient is calculated using Eq. 2. We compute the values of $Y^{\prime }$ by using the co-efficient values $\vec{b}$ in Eq. 1. Then the AUC for the pair of {*Y*, $Y^{\prime }$} is calculated. This AUC value indicates the level of influence of the corresponding pair of features in classifying the dataset. The feature set with the highest AUC value is selected as the node of the tree. Algorithm 1 presents pseudo code for selecting the most influential feature paired with another feature that has the highest AUC value, as a node in building the decision tree.

**Algorithm 1**: *selectGenes*: Selects the best combination of gene features based on AUC

**Input**: the training dataset: **D**, desired class labels **Y**, the best AUC value so far: bestAUC, the set of gene features that has generated bestAUC: GENE, the maximum number of gene features to be mapped onto a single value: limit

Comment(for *BVROC-tree *limit = 2)

**Output**: the derived single feature values: $Y^{\prime }$, the set of the features that generates the best AUC: selectedGenes, the best AUC: bestAUC

**if ***|GENE| >*= *limit ***then**

  selectedGenes = *GENE*;

  bestAUC = bestAUC;

  $Y^{\prime }$ = the $Y_{i}^{\prime }$ value obtained for selectedGenes;

  return;

end

**if ***GENE=*∅ **then**

  **for ***each gene feature ***A_i _***in ***D do**

    calculate $AUC_{A_{i}}$ for the pair of {**A_i_**, **Y**};

  **end**

  bestAUC = *max*($AUC_{A_{i}}$);

  *GENE *= the ith gene feature **A_i _**that generates bestAUC;

else

  **for ***each gene feature ***A_i _***paired with the gene feature or gene feature set GENE ***do**

    **if A_i _***is not in GENE ***then**

      *D_s _*= The dataset of all values for the gene features: **A_i _***∪ gene*;

      $\vec{b}\;=\;{\left(D_{s}^{T}D_{s}\right)}^{-1}D_{s}^{T}Y$;

      $Y_{i}^{\prime }={\vec{b}}^{T}\times \;D_{s}$;

      calculate $AUC_{{Y^{\prime }}_{i}}$ for the pair of $\left\{{Y^{\prime }}_{i},\;Y\right\}$;

    **end**

  **end**

  bestAUC = *max*($AUC_{{Y^{\prime }}_{i}}$);

  selectedGenes = the gene features **A_i _**∪ *GENE *that generate bestAUC;

  $Y^{\prime }$ = the $Y_{i}^{\prime }$ value obtained for gene features **A_i _**∪ *gene*;

end

[$Y^{\prime }$, selectedGenes, bestAUC] = SELECTGENES(**D**, **Y**, bestAUC, selectedGenes, limit);

Based on selection of a suitable splitting threshold (to be discussed shortly) for the selected set of genes, the dataset is then divided into two subsets. Each of the subsets is then used to further induce the tree in a similar way.

**Example 2 ***Let us consider a dataset D of m examples, where each example comprises k gene features: A*_1_, *A*_2_, *A*_3_, ..., *A_k_. Each of the k features has a differing discriminative power reflected by its respective AUC. Initially, to calculate the discriminative power that is expressed in terms of AUC, we compute the AUC for each gene feature paired with the desired class labels Y. The feature A_α _that produces the maximum AUC is selected. The selected gene feature A_α _is paired with each of the remaining features and for each pair the corresponding linear co-efficients are calculated. Using the linear co-efficients and Eq. 1 the set of class labels are predicted. For each set of predicted class labels the AUC value is computed. Suppose that the feature A_β_, where *1 *≤ β ≤ k and β ≠ α paired with the initially selected feature Aα. Consider *$\vec{b}$*(where *$\vec{b}=\left\langle b_{\alpha }\;,\;b_{\beta }\right\rangle$*) is the set of linear co-efficients for this pair of features *{*A_α_, B_β_*}. *The predicted class labels are *$Y_{\alpha ,\beta }^{\prime }$, *where*

$$Y_{\alpha ,\beta }^{\prime }=\vec{b}\;\;\times \;\;{\left(A_{\alpha }\;\;\;\;\;A_{\beta }\right)}^{T}$$

*The AUC_α,β _is calculated for the pair of *{*Y*, $Y_{\alpha ,\beta }^{\prime }$}. *If, AUC_α,β _is the maximum value among the AUCs for all other features (each of which is paired with the selected feature A_α_) and if, AUC_α,β _> AUC_α _then the set of features *{*A_α_, A_β _*} *is selected as the node. If, AUC_α,β _≤ AUC_α _the feature A_α _is selected as the node. A suitable threshold is then obtained for this feature and the dataset D is divided into two subsets: D_left _and D_right_. Then, for each subset D_left _and D_right_, we recursively use the similar process by excluding the features used at the parent nodes and thus, induce the decision tree*.

### Splitting threshold

Splitting threshold is the value of the selected feature that discriminates the classes. This is an important step in inducing the tree to select the best threshold value such that misclassification of instances is minimum. To select the splitting threshold we use the HRL function [4,5] in our *BVROC-tree*. The HRL is a loss function which measures the loss or degree of error of a given classifier's output for a splitting threshold value. Consider a classifier whose output is real numbers. Assume that there are 8 data instances and the corresponding classified outputs by the classifier are: $\left\langle -\text{1},\;-0.\text{4},\;-0.\text{7},\;-0.\text{9},\;0.0\text{1},\;0.\text{5},\;0.\text{9},\;\text{1}\right\rangle$, while the desired class labels for the corresponding instances are $\left\langle -1,\;-1,\;+1,\;-1,\;-1,\;-1,\;+1,\;+1\right\rangle$. The outputs are ranked according to the values as in Table 1:

**Table 1:** 

Output in actual ordering:	-1	-0.4	-0.7	-0.9	0.01	0.5	0.9	1
Output (sorted):	-1	-0.9	-0.7	-0.4	0.01	0.5	0.9	1

Rank:	1	2	3	4	5	6	7	8

Desired Output:	-1	-1	+1	-1	-1	-1	+1	+1

For a threshold value *θ*; the data instances are labeled either as -1 or +1 using classifier output as follows:

$$ClassLabel\left(Predicted\right)\text{ = }\left\{\begin{array}{cc} -\text{1}, & \text{if}\;Output\leq \theta ;\\ +\text{1}, & \text{if}\;Output>\theta . \end{array}\right)$$

Considering *θ *= -0.4, the labeling of the data instances are obtained as in Table 2:

**Table 2:** 

Output (sorted):	-1	-0.9	-0.7	-0.4	0.01	0.5	0.9	1
	
Rank:	1	2	3	4	5	6	7	8
	
Desired Output:	-1	-1	+1	-1	-1	-1	+1	+1
	
Predicted Class Label:	-1	-1	-1	-1	+1	+1	+1	+1
	
*TN*:	✓	✓		✓				
	
*FP*:			✓					
	
*TP*:							✓	✓
	
*FN*:					✓	✓		
	
*Rankdistancepenalty*:	0	0	2	0	1	2	0	0

In the HRL function, if the classifier's output is greater than *θ *for an instance whose desired class label is -1 then it is counted as false positive (FP), otherwise it is a true negative (TN). Similarly, for a data instance with a desired class label +1, if the classifier's output is less than or equals to *θ *then it is a false negative (FN), otherwise it is a true positive (TP). The rank distance penalty for each FN or TP instance corresponds to its distance in terms of ranks from the threshold. The HRL is the sum of all rank distance penalties. In the above example the total penalty for the false positives is 2 and the total penalty for the false negatives is 1 + 2 = 3, and the overall HRL is 2 + 3 = 5 [5].

In selecting the splitting threshold, we attempt to classify the dataset considering each value in the $Y^{\prime }$ ($Y^{\prime }$ is obtained for the feature computed through linear combination of more than one gene). For each chosen value *θ *the corresponding HRL is computed. The *θ *that generates the minimum HRL is selected as the splitting threshold.

**Example 3 ***Let us consider the dataset D of m instances, where each instance has k features: A*_1_, *A*_2_, *A*_3_, *..., A_k_. Suppose, the linear combination of the pair of features *{*A_α_, A_β_*}, *where *1 *≤ α ≤ k*, 1 *≤ β ≤ k and α ≠ β, has the highest AUC and is selected to be the node in the tree. The set of features *{*A_α_, A_β_*} *is projected to a set of single values *$Y_{\alpha ,\beta }^{\prime }$*where *$\left\{{y^{\prime }}_{1},{y^{\prime }}_{2},{y^{\prime }}_{3},\;.\;.\;.\;,{y^{\prime }}_{m}\right\}\in Y_{\alpha ,\beta }^{\prime }$*Each value of *$Y_{\alpha ,\beta }^{\prime }$*is sorted and ranked. Let us rank the values without loss of generality as in Table *3:

**Table 3:** 

$Y_{\alpha ,\beta }^{\prime }$:	$y_{1}^{\prime }$	$y_{2}^{\prime }$	$y_{3}^{\prime }$	...	$y_{m}^{\prime }$
Rank:	1	2	3	...	m

*Then for each value *$y_{1}^{\prime },y_{2}^{\prime },y_{3}^{\prime },\;.\;.\;.\;,y_{m\;}^{\prime }$*of *$Y_{\alpha ,\beta }^{\prime }$, *we form the rule*:

$$Class\left(Predicted\right)=\left\{\begin{array}{cc} -\text{1}, & if\;Y_{\alpha ,\beta }\leq \;y_{j}^{\alpha };\\ +\text{1}, & Otherwise. \end{array}\right)$$

*where *1 *≤ j ≤ m. We then attempt to classify the dataset with this rule, and note the HRL*.

*Then the value *$y_{j}^{\alpha }$*with the minimum HRL is selected as the splitting threshold for the pair of features *{*A_α_, A_β_*}.

Pseudo code for calculating the splitting threshold is presented in Algorithm 2.

### Stopping criterion

To stop growing the tree, the AUC of the selected combination of genes is tested. If the AUC value is equal to 1, yields that the combination of genes can classify the training dataset accurately with 100% *sensitivity *and 100% *specificity*. Therefore, there is no need to grow the tree further at this node. However, to avoid over fitting, we choose an AUC value *≥ *0.95 in order to stop growing the tree for a node. This facilitates us not to grow the tree for a smallest subset of the training dataset.

### Labeling the leaf nodes

Each leaf node is labeled with a class label which is obtained by the majority of the class instances in that node. Algorithm 3 presents the pseudo code for inducing the *BVROC-tree *using the functions presented in Algorithm 1 and Algorithm 2.

## Related work

Several methods for constructing multivariate decision trees exist. In this section we describe some of the existing multivariate decision trees.

Linear discriminant analysis(LDA) has been used to combine multiple features at each node of the decision tree known as linear discriminant tree (LDT) developed by [6]. In this process, the impurity measurement is same as the C4.5 except that the splitting of combination of feature is done using LDA. It is claimed that the LDA based multivariate decision

**Algorithm 2**: CalculateSplitThreshold

**Input**: *Y *: The actual class label, $Y^{\prime }$: The derived value from more than one gene feature

**Output**: *θ*: Splitting threshold for the node

*HRL *= ∅; *θ *= ∅;

Sort and rank $Y^{\prime }$;

**for ***each rank r ***do**

  *splitThreshold *= the value of $Y^{\prime }$ that corresponds to *r*;

  **D_neg _**= ∅; **D_pos _**= ∅;

  **for ***each *$Y^{\prime }$**do**

    **if **$Y_{i}^{\prime }$*≤ splitThreshold ***then**

      **D_neg _**= **D_neg _**∪ {$Y_{i}^{\prime }$, *Y_i_*}

    **end**

    **D_pos _**= **D_pos _**∪ {$Y_{i}^{\prime }$, *Y_i_*}

  **end**

  *TotalHRL *= ∅;

  **for ***each Y_i _in ***D_neg _do**

    **if ***Y_i _*= +1 **then**

      *TotalHRL *= *TotalHRL *+ *|D_neg_| − i *+ 1;

      Comment: *|D_neg_| *represents the total number of instances in *D_neg _*and the value of *i *ranges from 1 to |**D_neg_**|;

    **end**

  **end**

  **for ***each Y_i _in ***D_pos _do**

    **if ***Y_i _*= -1 **then**

      *TotalHRL *= *TotalHRL *+ *i*;

      Comment: the value of *i *ranges from 1 to |**Dpos**|;

    **end**

    **if ***TotalHRL < HRL ***then**

      *HRL *= *TotalHRL*;

      *θ *= *splitThreshold*;

    **end**

  **end**

end

return *θ*;

tree can learn faster than other multivariate trees, however, the classification performance is no better than the other multivariate trees.

Breiman *et al. *[1] first introduced Classification And Regression Tree abbreviated CART, where multiple features are combined at a node of the tree. The algorithm looks for a splitting point followed by a linear test that achieves the least impurity. The limitation of CART is that, it can get stuck in a local minimum since the algorithm stops searching for the further combination of features when the impurity gets an increase in next to the current execution of above process. However, OC1 [7] a variant of CART solves this problem where the parameter update method follows the CART method, but includes random perturbations

**Algorithm 3**: *BVROC-tree*

**Input**: The matrix of training examples: D ; the vector of class labels: *Y*

**Output**: *T*: A *BVROC-tree *decision tree

**if **D=∅**then**

  return a single node with ∅;

end

**if ***Y consists of records all with the same value for the class label ***then**

  return a single leaf node with that value;

end

[$Y^{\prime }$, GeneSet, AUC] = SELECTGENES(**D**, **Y**, 0, ∅, 2);

*θ *= CALCULATESPLITTHRESHOLD(*Y*, $Y^{\prime }$);

Assign $D_{left}$ and $D_{right}$ as the subsets of D  consisting of records respectively with the value greater than or equal to and less than *θ*;

Assign *Y_left _*and *Y_right _*as the subsets of *Y *that correspond to the examples in $D_{left}$ and $D_{right}$ respectively;

Recursively apply *BVROC-tree *to subsets {$D_{left}$, *Y_left_*} and {$D_{right}$, *Y_right_*} until they are empty or the stopping criteria are met; return a tree T  with root or node labeled A  and arcs labeled *a*_1 _and *a*_2_, going respectively to the trees *BVROC-tree(*$D_{left}$, *Y_left_) and BVROC-tree(*$D_{right}$, *Y_right_);*

of the parameters when a local minimum is reached and restarts from random location.

Logistic Model Tree (LMT) is another multivariate decision tree developed by [8], where features are combined using linear logistic regression and the selection of features and splitting are done following the same process as in C4.5.

Our *BVROC-tree *described in this paper is different to all other existing trees in that we use a novel method based on AUC and the linear mapping function (using least square estimation) to select the combination of features to form a node. We also use a splitting criteria based on HRL as was used in *ROC-tree *[4](the predecessor of our *BVROC-tree*).

## Experimental setup and datasets

For the experimental analysis, we compare against a number of well known simple decision tree induction techniques: *ROC-Tree *a predecessor of the proposed method, C4.5 [9], Ferri *et al*.'s [10] AUCsplit technique for decision trees, ADTree [11], Random Forest [12], REPTree and Random Tree. We also compare against the non-decision tree classifiers: Naïve Bayes and *k*-NN.

### Datasets and validation scheme

Each of the techniques is applied on seven gene expression datasets. The properties of the datasets are illustrated in Table 1. To evaluate the performance of *BVROC-Tree*, a 10-fold cross validation (CV) scheme is used 5 times for all datasets.

**Table 4:** Datasets.

Dataset	Data collected from	No. of genes	Total Samples	Classification of:
GE1	Critchley-Thorne *et al. *[13]	20,845	46	Metastatic Melanoma

GE2	Zizhen *et al. *[14]	4133	101	Marfan Syndrome

GE3	Gordon *et al. *[15]	12,533	181	Lung Cancer

GE4	Singh *et al. *[16]	12,600	21	Prostate cancer

GE5	Singh *et al. *[16]	12,600	136	Prostate cancer

GE6	Golub *et al. *[17]	7,129	72	Leukemia

GE7	Notterman *et al. *[18]	22,278	19	Colorectal Adenoma

Properties of the datasets used in this study

## Results and discussion

The classification accuracies for all techniques on the considered gene expression datasets are presented in Table 5. The classification performances in AUC are presented in Table 6. In each table, the best performances among that of the reported classifiers are marked in bold.

**Table 5:** Performance in accuracy.

Method	GE1	GE2	GE3	GE4	GE5	GE6	GE7
BVROC-Tree	**66.85 **± **3.26**	84.16 ± 0.02	**98.9 **± **0.90**	**52.38 **± **15.06**	**89.95 **± **3.31**	**97.57 **± **1.33**	**77.97 **± **0.03**
ROC-Tree	64.13 ± 4.53	86.26 ± 0.05	98.34 ± 0.89	38.10 ± 5.95	88.24 ± 2.33	94.44 ± 2.96	52.63 ± 0.07
AUCsplit	56.96 ± 0.09	81.93 ± 0.02	96.14 ± 1.36	34.01 ± 2.87	82.47 ± 3.96	81.61 ± 3.28	50.53 ± 0.07
C4.5	53.48 ± 5.67	78.04 ± 1.83	93.21 ± 1.07	41.7 ± 4.74	79.42 ± 5.45	84.39 ± 2.01	39.00 ± 5.48
ADTree	55.22 ± 5.87	**89.89 **± **2.80**	95.14 ± 2.17	43.10 ± 4.80	86.76 ± 2.63	88.82 ± 5.06	49.00 ± 4.18
REPTree	58.26 ± 2.83	78.64 ± 2.99	95.01 ± 1.79	44.23 ± 5.18	80.88 ± 3.33	87.64 ± 4.49	57.00 ± 13.51
Random Tree	51.74 ± 1.82	65.53 ± 3.24	92.03 ± 5.62	46.40 ± 6.74	62.50 ± 5.23	81.64 ± 11.47	47.00 ± 16.43
Random Forest	48.6 ± 4.85	81.45 ± 4.62	92.98 ± 5.36	47.52 ± 7.19	80.88 ± 2.56	82.13 ± 10.33	43.00 ± 10.37
Naïve Bayes	50.60 ± 5.82	88.60 ± 2.26	93.85 ± 5.27	46.15 ± 7.44	55.88 ± 4.76	84.85 ± 11.26	62.00 ± 4.47
*k*-NN	47.10 ± 5.31	86.80 ± 2.29	93.73 ± 4.88	48.23 ± 8.61	78.68 ± 4.78	84.68 ± 10.42	44.00 ± 4.18

Table representing the overall accuracy for gene expression datasets using 5 × 10 fold cross-validation scheme

**Table 6:** Performance in AUC.

Method	GE1	GE2	GE3	GE4	GE5	GE6	GE7
BVROC-Tree	**0.69 **± **0.04**	0.82 ± 0.04	**0.93 **± **0.03**	**0.49 **± **0.22**	**0.89 **± **0.04**	**0.97 **± **0.01**	**0.77 **± **0.06**
ROC-Tree	0.64 ± 0.09	0.79 ± 0.05	0.93 ± 0.04	0.29 ± 0.05	0.89 ± 0.33	0.95 ± 0.01	0.54 ± 0.08
AUCsplit	0.57 ± 0.10	0.78 ± 0.02	0.92 ± 0.02	0.30 ± 0.06	0.81 ± 0.04	0.82 ± 0.08	0.49 ± 0.11
C4.5	0.56 ± 0.05	0.78 ± 0.03	0.87 ± 0.03	0.39 ± 0.04	0.78 ± 0.06	0.83 ± 0.02	0.45± 0.05
ADTree	0.57 ± 0.04	**0.96**± **0.02**	0.92 ± 0.06	0.36 ± 0.05	0.84 ± 0.03	0.90 ± 0.08	0.50 ± 0.06
REPTree	0.59 ± 0.06	0.80 ± 0.02	0.91 ± 0.05	0.40 ± 0.07	0.79 ± 0.04	0.88 ± 0.07	0.61± 0.08
Random Tree	0.55 ± 0.03	0.64 ± 0.04	0.85 ± 0.12	0.43 ± 0.09	0.63 ± 0.05	0.81 ± 0.14	0.53 ± 0.15
Random Forest	0.54 ± 0.05	0.89 ± 0.04	0.88 ± 0.12	0.43 ± 0.09	0.79 ± 0.03	0.83 ± 0.13	0.47 ± 0.21
Naïve Bayes	0.55 ± 0.05	0.93± 0.02	0.89 ± 0.12	0.42 ± 0.09	0.53 ± 0.05	0.86 ± 0.14	0.65 ± 0.11
*k*-NN	0.53 ± 0.03	0.93 ± 0.02	0.91 ± 0.11	0.42 ± 0.09	0.79 ± 0.05	0.87 ± 0.13	0.51 ± 0.09

Table representing the AUC result for gene expression datasets using 5 × 10 fold cross-validation scheme

### Classification performance of *BVROC-tree*

The classification performance of *BVROC-Tree *on the gene expression datasets clearly outperforms that of all the other reported decision trees (see Table 2). *ROC-Tree*, the predecessor of *BVROC-Tree*, have been reported in a previous study to perform consistently better classification in terms of accuracy and AUC measurement compared with other variants of decision tree classifiers including C4.5. Interestingly, the classification performance of *BVROC-Tree *is even better than its predecessor *ROC-Tree*. More specifically, for the datasets GE4 and GE7 this performance improvement of the *BVROC-tree *is respectively at least 37% and 48% better than the *ROC-tree*. Furthermore, the performance improvement of the *BVROC-tree *over the other best performing decision trees is at least 17%, 3%, 10%, 4%, 10% and 37% for the datasets GE1, GE3, GE4, GE5, GE6, and GE7 respectively. This is evident that one of the reasons for this better performance is due to the application of more than one feature at each node of the tree along with the better computation of discriminative power of features used when building the tree and better splitting criteria that balances the loss and gain.

### Comparison of AUC values

We also computed the overall AUC value of all classifiers considered in this paper (see Table 3), resulting from the 5 × 10-fold cross validation over the gene-expression datasets. The AUC values of *BVROC-tree *is as good as of *ROC-tree *for the datasets GE3, GE5 and GE6. For the other datasets the AUC values of *BVROC-tree *are much higher than that of *ROC-tree*. As the classification accuracy, the AUC values of ADTree for datasets GE2 is the best among all classifiers. However, for other datasets *BVROC-tree *and its predecessor *ROC-tree *outperform ADTree. Specifically, for six of the seven gene expression datasets, we see the *BVROC − tree *has better AUC than other classifiers.

### Comparison of tree sizes

The size of each tree built using the *BVROC-tree *method always smaller compared to the other decision trees for all the datasets considered in this paper. We see in Table 4 that, the range of the size of *BVROC-tree *is in between 2 to 3. While this range for C4.5 is in between 3 to 39. Although the range of the size of *REPTree *is from 1 to 51, the performance of *REPTRee *is much lower than the performance of *BVROC-tree *(see Table 7). Since, in *BVROC-tree*, a maximum two features are used to form a node, the range of features used in inducing *BVROC-tree *is from 4 to 6 features. The combination of multiple features at each node using linear mapping can achieve a better discriminant strength compared to the single feature, and hence using a smaller tree size as induced in *BVROC-tree*, a better classification performance is achieved when compared to the other decision trees.

**Table 7:** Tree size.

	Tree size					
	
	*BVROC-tree*	*ROC-tree*	C4.5	ADTree	REPTree	Random Tree
GE1	3	5	7	28	4	52

GE2	2	6	5	22	3	18

GE3	3	7	7	26	4	61

GE4	2	7	5	30	3	27

GE5	3	16	10	32	5	86

GE6	2	6	4	31	3	42

GE7	2	5	3	28	1	21

Comparison of the sizes of the trees using all the data instances as training data

## Conclusion

We proposed a new decision tree *BVROC-tree *which considers more than one feature at each node. The selection of features at each node makes use of linear mapping of multiple features to obtain a derived feature whose AUC values can be easily computed. Our experimental results show that the *BVROC-tree *outperforms several state of the art competing classifiers both in accuracy and AUC values. Our experimental results show that our method is very effective for gene expression data with high number of dimensions. We believe that our proposed algorithm is a very practical and useful solution in classifying gene expression data. Since the classification accuracy has not been achieved as 100%, there exist scopes to enhance the *BVROC-tree*. In *BVROC-tree *we restricted the algorithm to combine a maximum of two features at each node of the tree. The classification performance could be improved by combining more than two features at each node, however in this case an intelligent method must be introduced such that the computational complexity remains reasonable. We plan to replace the linear mapping with any existing non-linear mapping (e.g., through application of polynomial kernel or gaussian kernel) of multiple features as a node of the decision tree with an aim to further enhance the performance of the *BVROC-tree*.

## Competing interests

MRH has received grant from Deanship of Scientific Research, King Fahd University of Petroleum and Minerals in conducting the research. RK received support from the University of Melbourne for the cost of the publication. All authors declare that there is no financial relationships other than the above mentioned ones with any organisations that might have an interest in the submitted work in the previous three years; no other relationships or activities that could appear to have influenced the work described in this paper.

## Authors' contributions

The idea of the *BV-ROC *was generated by MRH. MRH implemented the method and prepared the manuscript. RK helped in preparing the manuscript.

## Declarations

Publication of this work was supported by Ramamohanarao Kotagiri's funding at the University of Melbourne.

This article has been published as part of *BMC Proceedings *Volume 7 Supplement 7, 2013: Proceedings of the Great Lakes Bioinformatics Conference 2013. The full contents of the supplement are available online at http://www.biomedcentral.com/bmcproc/supplements/7/S7.
